# *Hj*‐aggregates of quinoline‐fused boron‐dipyrromethene nanoparticles for near‐infrared dualmodal photodynamic and photothermal therapy

**DOI:** 10.1002/smo2.70097

**Published:** 2026-08-23

**Authors:** Bo Zhang, Chuang Du, Yuewen Yang, Dongping Xu, Xin‐Dong Jiang, Hui‐Xin Liu, Gaowu Qin

**Affiliations:** ^1^ Institute for Strategic Materials and Components Shenyang University of Chemical Technology Shenyang China; ^2^ Institute of Life Sciences China Medical University Shenyang China; ^3^ Department of Gastroenterology Shengjing Hospital of China Medical University Shenyang China

**Keywords:** BODIPY, *Hj*‐aggregate, quinoline‐fused pyrrole, ROS

## Abstract

Development of boron‐dipyrromethene (BODIPY)‐based phototherapeutic agents integrating photodynamic and photothermal therapy is limited by restricted modifiable sites on traditional scaffolds. Herein, we report a quinoline‐fused cyclic pyrrole Npy generated by an lithium diisopropylamide‐catalyzed [3 + 2] cyclization, which incorporates a nitrogen atom into a rigid polycyclic π‐system. Using this pyrrole Npy, a symmetric photosensitizer Npy‐BDP with absorption at 660 nm was prepared with a singlet oxygen quantum yield of 0.38, and efficient type‐I reactive oxygen species generation. Upon nanoparticle encapsulation, Npy‐BDP forms *Hj*‐aggregates with absorption peaks at 562 and 703 nm. Npy‐BDP NPs achieve a photothermal conversion efficiency of 53% and generate local hyperthermia under 690 nm laser irradiation. In vitro Npy‐BDP NPs exhibit potent photocytotoxicity against HeLa cells with low dark toxicity and induce apoptosis through the mitochondrial pathway. In vivo intratumoral injection of Npy‐BDP NPs followed by laser irradiation resulted in sustained tumor regression. This work presents the first example of *Hj*‐aggregates in the BODIPY system, providing a new molecular design paradigm for phototherapy reagents.

## Graphical abstract

A novel quinoline‐fused cyclic pyrrole is synthesized via lithium diisopropylamide‐catalyzed [3 + 2] cyclization, enabling the construction of a symmetrical boron‐dipyrromethene photosensitizer that forms *Hj*‐aggregates upon nanoparticle encapsulation.

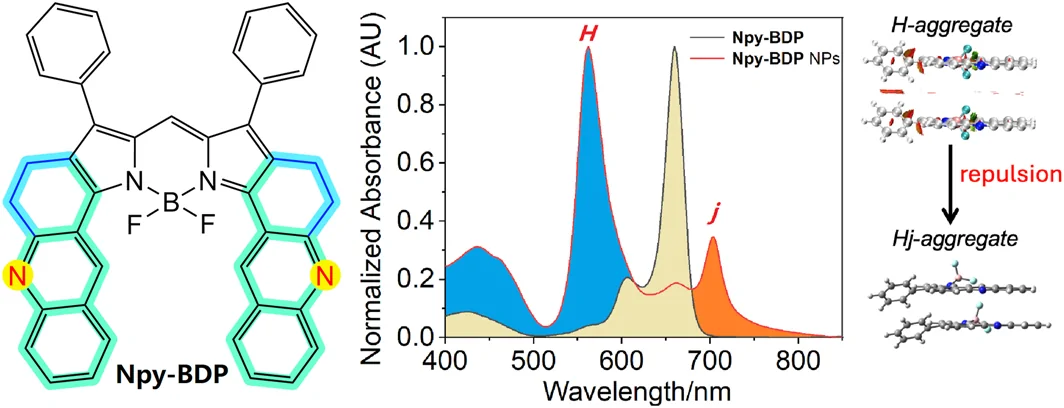

## 1 INTRODUCTION

**FIGURE 1:**
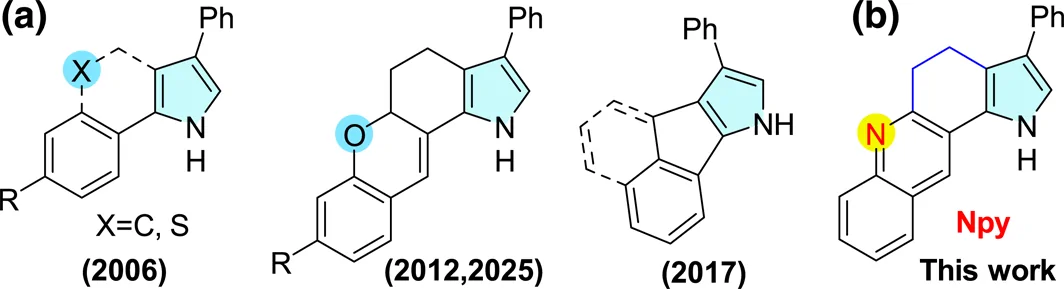
Cyclic‐fused pyrrole structure. (a) Early traditional pyrrole scaffolds. (b) Nitrogen‐containing pyrrole scaffold.

Boron‐dipyrromethene (BODIPY) dyes with high molar extinction coefficient, tunable wavelength, and ease of functionalization, have established themselves as a versatile near‐infrared (NIR) platform.[^1^, ^2^, ^3^] Traditional strategies to redshift BODIPY absorption rely on extending the conjugation at peripheral sites of classic pyrrole core (Figure 1a) or *J*‐aggregates of monomer.[^4^, ^5^] However, conjugate strategies are intrinsically constrained by the limited availability of modifiable positions, often accompanied by complex synthetic routes, suboptimal photophysical properties, and inadequate phototherapeutic efficacy,[^6^, ^7^, ^8^, ^9^] all of which remain critical challenges to be resolved.^[10]^ Moreover, achieving concurrent enhancements in therapeutic parameters, such as intersystem crossing (ISC) for photodynamic (PDT) and non‐radiative decay for photothermal therapy (PTT), within a simple synthetic single‐molecule structure remains rare.[^11^, ^12^, ^13^] Therefore, structural innovation of pyrrole is crucial for developing functionalized BODIPYs.

In 2005, Zhao and Carreira first reported a synthetic strategy for the efficient construction of pyrroles, enabling the synthesis of the rigid 2,4‐diarylpyrroles (Figure 1a).^[14]^ This methodology involves the ring‐forming reaction between 3‐phenyl‐2*H*‐azirine and ketone under basic conditions, providing access to conformationally constrained pyrrole scaffolds. Especially, conventional ring‐fused pyrroles are typically composed of benzene rings and cycloalkanes. To the best of our knowledge, no *N*‐heteroatom containing pyrrole is known to date.[^15^, ^16^, ^17^] Hence, this work provides the paradigm through a fundamental innovation of crucial intermediate pyrrole.

BODIPY dye is known to assemble into *H*‐ or *J*‐aggregates, primarily characterized by aggregation‐induced spectral shifts that align with Kasha theory.[^18^, ^19^] Charge transfer (CT) interactions introduce short‐range superexchange coupling, which can compete with Coulombic interactions but exhibit distinct dependencies on intermolecular geometry. The concurrent presence of these dual coupling mechanisms enables a more diverse spectrum of aggregate types, denoted as *HJ*, *HH*, *JH*, and *JJ*. In this notation, the first letter denotes the dominant contribution from Coulombic coupling, while the second letter indicates the influence of CT‐mediated coupling. Lowercase and uppercase lettering may be employed to signify the relative magnitudes of these respective interactions. To the best of our knowledge, there have been no reports of *Hj*‐aggregates in the BODIPY system. Herein, we have systematically gained insights into the optical properties of *Hj*‐aggregates of Npy‐BDP nanoparticles (Npy‐BDP NPs) for the first time.

**SCHEME 1:**
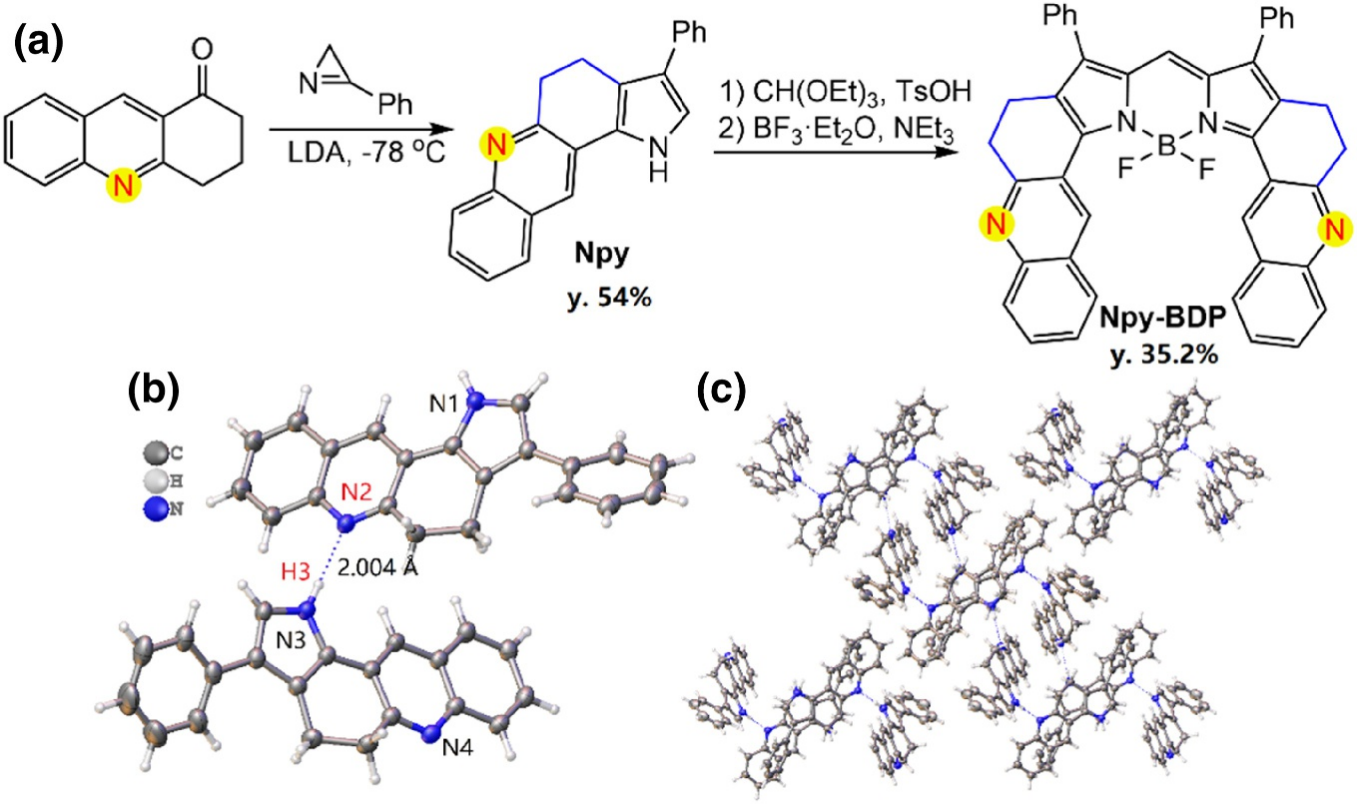
(a) Synthetic route to pyrrole Npy and boron‐dipyrromethene Npy‐BDP. (b) X‐ray crystallographic analysis of pyrrole Npy (CCDC: 2551150). (c) Single crystal stacking diagram of pyrrole Npy.

So, extending the π‐conjugation by ring fusion, heavy‐atom‐free nitrogen atom was incorporated into the fused framework, and quinoline‐fused pyrrole Npy was prepared for the first time (Figure 1b). By LDA‐mediated [3 + 2] cyclization between 3,4‐dihydroacridin‐1(2*H*)‐one and 3‐phenyl‐2*H*‐azirine,[^20^, ^21^] pyrrole Npy possesses an intrinsically rigid and twisted polycyclic system with a –CH_2_–CH_2_– bridge and embedded *N* atom. Utilizing this pyrrole Npy, a symmetrical Npy‐BDP photosensitizer was obtained (Scheme 1). It achieved a significant absorption/emission redshift into the therapeutic NIR‐I window (*λ*_abs_/*λ*_em_ = 660/676 nm) with a remarkably high molar extinction coefficient (*ε* = 155,000 M^−1^ cm^−1^), which was crucial for deep‐tissue penetration and light harvesting. The heteroatom effect and twisted structure synergistically enhance ISC, endowing the unique capability to generate reactive oxygen species (ROS), which is a critical feature for overcoming hypoxia in tumors. The self‐assembled *Hj*‐aggregates of Npy‐BDP NPs exhibited two absorption peaks at 562 and 703 nm in the NIR region. Efficient non‐radiative decay was concurrently facilitated, providing photothermal conversion efficiency (*η* = 53%), thereby enabling potent PTT under a 690 nm NIR laser irradiation.

Moreover, Npy‐BDP NPs as heavy‐atom‐free photosensitizer efficiently generate both localized hyperthermia and substantial ROS, leading to effective apoptosis in cancer cells.^[22]^ This dye combines exceptional photostability, and dual PDT/PTT functions in a symmetric structure, as conclusively validated by significant tumor suppression in both cellular and murine models. Herein, reinventing the core heterocyclic architecture in pyrrole, is a powerful and necessary strategy to achieve innovative photo‐response agents of *Hj*‐aggregates.

## 2 RESULTS AND DISCUSSION

To introduce an *N*‐heteroatom into the pyrrole ring, 3,4‐dihydroacridin‐1(2*H*)‐one was employed as the starting material. In the presence of lithium diisopropylamide (LDA) and 3‐phenyl‐2*H*‐azirine, a quinoline‐fused cyclic pyrrole Npy was efficiently synthesized in a one‐pot reaction (Scheme 1a).^[23]^ This synthetic route is operationally simple, directly delivering a rigid polycyclic π‐system that serves as an ideal precursor for constructing functional BODIPY dyes. The structure of Npy was unambiguously confirmed by ^1^H nuclear magnetic resonance (NMR) spectroscopy, which displayed a characteristic broad singlet at *δ* = 9.24 ppm corresponding to the *N*–*H* proton of the pyrrole (Figure S1). The solid‐state structure of Npy (CCDC: 2551150) was further confirmed by single‐crystal X‐ray diffraction analysis. The crystal structure clearly revealed that the flexible –CH_2_–CH_2_– chain was covalently locked within the pyrrole framework, resulting in a rigid, ring‐fused and twisted conformation. Furthermore, crystal packing analysis (Scheme 1b) indicated the presence of an intermolecular hydrogen bond (*N3–H3*···*N2* distance: 2.004 Å). These strong interactions enhance the structural stability in the solid state and promote ordered molecular stacking, suggesting the potential of such compounds as novel organic functional materials (Scheme 1c).^[24]^ Using this pyrrole Npy, the symmetric dye Npy‐BDP was successfully prepared by a straightforward one‐step bridging strategy (Scheme 1a). The molecular structure of Npy‐BDP was comprehensively characterized by ^1^H NMR, ^13^C NMR, and high‐resolution mass spectrometry (Figures S2–S5).

**FIGURE 2:**
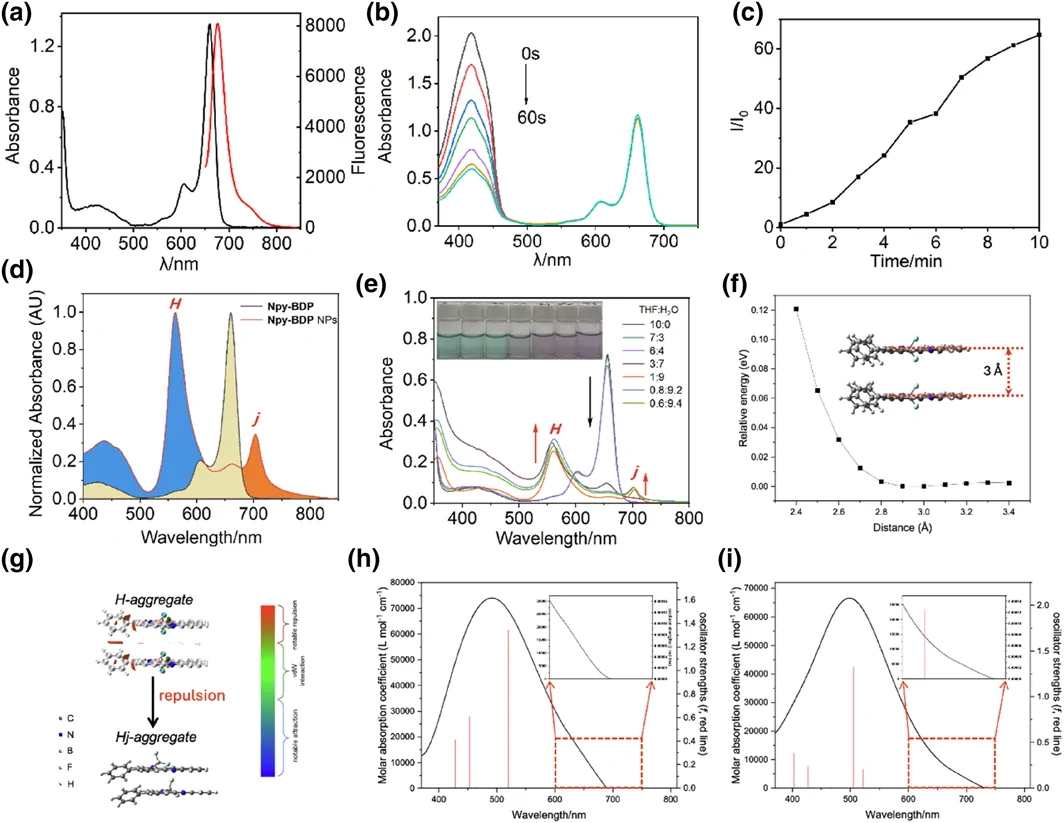
(a) Absorption spectra (red) and emission spectra (purple) in CH_2_Cl_2_ of Npy‐BDP at 298 K. (b) Time‐dependent photodegradation of 1,3‐diphenylisobenzofuran with Npy‐BDP (690 nm, 0.3 W/cm^2^). (c) ROS generation of Npy‐BDP (10 μM) detected by DHR123 (10 μM) in DMSO under 690 nm laser (0.1 W/cm^2^, 10 min). (d) Normalized absorbance of Npy‐BDP in CH_2_Cl_2_ (black curve) and Npy‐BDP NPs (red curve) in aqueous solution. (e) Absorption changes of Npy‐BDP in THF/H_2_O with varying water fractions. (f) Potential energy surface scan along the intermolecular distance between two Npy‐BDP molecules in the *H*‐aggregate. (g) Reduced density gradient analysis of the *H*‐aggregate (top) and the optimized structure of *Hj*‐aggregate (bottom). UV‐Vis absorption spectra obtained from TDA‐TDDFT calculations for the (h) *H*‐aggregates and (i) *Hj*‐aggregates at the ωB97X‐D3/6‐311+G(d,p) level, with the 600–750 nm region enlarged to highlight the light‐absorption peaks (Oscillator Strengths). DMSO, dimethyl sulfoxide; ROS, reactive oxygen species; THF, tetrahydrofuran.

Benefiting from the rigid *N*‐incorporated extended conjugation system derived from the pyrrole Npy, Npy‐BDP exhibited a pronounced redshift in both absorption and emission spectra. Compared to the non‐fused and fused‐ring BODIPY derivatives listed in Table S1,[^25^, ^26^, ^27^, ^28^, ^29^, ^30^, ^31^, ^32^, ^33^] Npy‐BDP showed a dramatic redshift in both absorption/emission (*λ*_abs_/*λ*_em_ = 660/676 nm) and narrow full width at half maximum (FWHM = 24 nm) (Figure 2a), extending deeper into the NIR biological window. The molar extinction coefficient (155,000 M^−1^ cm^−1^) is significantly higher than all the comparative dyes (Table S1), indicating enhanced light‐harvesting capability. The fluorescence quantum yield (Φ_f_ = 0.11) ensures that Npy‐BDP retains optical imaging functionality while facilitating the transition of excited‐state energy toward ISC, thereby promoting the generation of ROS and non‐radiative photothermal conversion. This balanced photophysical profile makes Npy‐BDP suited for dualmodal application, demonstrating the advantage of designing a high‐performance BODIPY photosensitizer from this pyrrole scaffold. Furthermore, absorption spectra in various organic solvents confirmed the excellent NIR spectral stability of Npy‐BDP, exhibiting only slight emission wavelength shifts (e.g., *λ*_em_ = 674 nm in tetrahydrofuran (THF), 681 nm in dimethyl sulfoxide (DMSO)) (Figure S6 and Table S2).

Next, the ROS generation capability of Npy‐BDP was evaluated. For type‐II ROS,^[34]^ the time‐dependent absorbance decay of 1,3‐diphenylisobenzofuran at 418 nm under 690 nm laser irradiation confirmed efficient ^1^O_2_ production (Figure 2b), with methylene blue (MB, Φ_Δ_ = 0.52 in DMSO) as the reference standard.^[35]^ A linear fit of the decay data (*R* ^2^ = 0.997) yielded a ^1^O_2_ quantum yield of Φ_Δ_ = 0.38 (Figure S7), due to the enhancement of ISC by the heteroatom in the Npy segment. Excellent photostability was concurrently evidenced by the stable absorption of Npy‐BDP at 660 nm during irradiation (Figure 2b). For type‐I ROS,[^36^, ^37^] the fluorescence signal of dihydrorhodamine 123 increased ∼65‐fold after 10 min of laser exposure, verifying efficient superoxide anion generation (Figure 2c). This is consistent with the design concept of heavy‐atom free‐type I photosensitizers recently reported, both of which enhance the electron transfer efficiency through intramolecular charge separation.^[38]^ Furthermore, Npy‐BDP nanoparticles in PBS can also induce ROS production under illumination (Figure S8). Therefore, these results demonstrate that Npy‐BDP operates as a photosensitizer, capable of simultaneously generating both type‐I and type‐II ROS upon light excitation, which exhibits its potential for advanced phototherapeutic applications.

**FIGURE 3:**
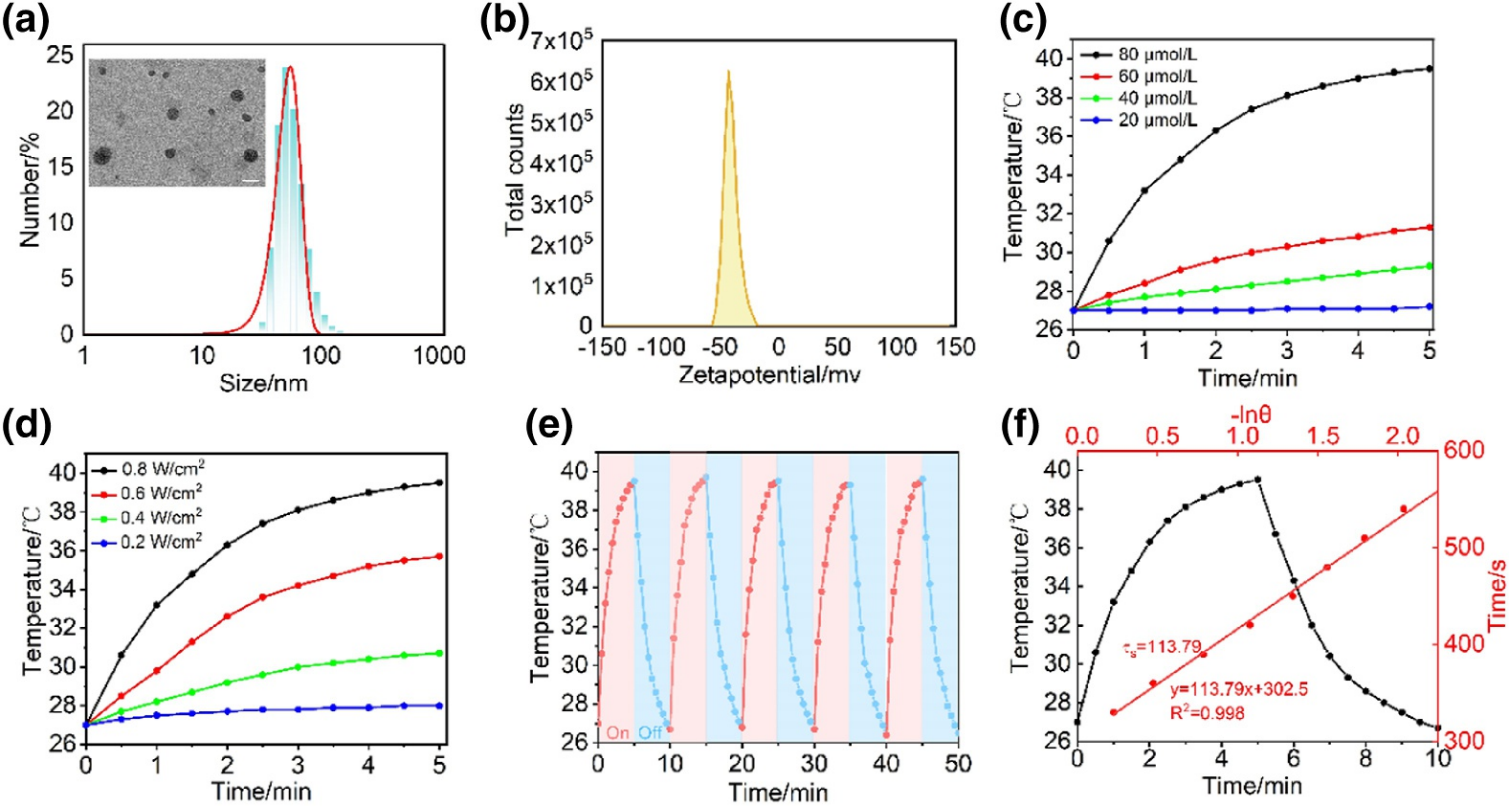
(a) DLS of Npy‐BDP NPs in aqueous solution. Inset image: TEM of Npy‐BDP NPs. Scale bar: 100 nm. (b) Zeta potential of Npy‐BDP NPs. (c) Temperature changes at various concentrations (20–80 μM) of Npy‐BDP NPs under 690 nm near‐infrared light excitation for 5 min (0.8 W/cm^2^). (d) Temperature variations at 80 μM concentrations for different laser power densities for 5 min (690 nm, 0.2–0.8 W/cm^2^). (e) Temperature variations over five heating/cooling cycles. (f) Photothermal response curves of Npy‐BDP NPs (80 μM) under irradiation and natural cooling, and linear fitting of −Ln*θ* and time. DLS, dynamic light scattering; TEM, transmission electron microscopy.

To enhance biocompatibility and targeting for biomedical applications, Npy‐BDP was encapsulated with DSPE‐PEG_2000_ by self‐assembly to form water‐soluble nanoparticles (Npy‐BDP NPs) (Figure 3a).^[39]^ Transmission electron microscopy and dynamic light scattering revealed that Npy‐BDP NPs possessed a uniform spherical morphology with hydrodynamic diameters of approximately 55.6 nm (Figure 3a).^[40]^ Npy‐BDP NPs also exhibited excellent colloidal stability in aqueous solution (Figure S9), as indicated by a uniform surface charge distribution with an average zeta potential near −42.9 mV (Figure 3b). Npy‐BDP NPs showed strong absorption at the 562 nm hypochromatic shift and weak absorption at 703 nm bathochromic shift (Figure 2d). In Npy‐BDP π‐stacks the larger Coulomb coupling, arising from the more efficient overlap between neighboring chromophores, tilts the scales in favor of *Hj*‐aggregates. Moreover, at a THF/H_2_O ratio of 3:7, Npy‐BDP mainly exhibited *H*‐aggregates (*λ*_abs_ = 566 nm); at higher water fractions, an additional peak at 704 nm was gradually enhanced, indicating the formation of *Hj*‐aggregates (Figure 2e). Additionally, the observation of multiple distinct lifetime components in time‐resolved fluorescence spectroscopy excluded the simple monomer/*H*‐aggregate co‐existence model. In conjunction with the bimodal absorption spectra, this unequivocally confirmed the formation of *Hj*‐type aggregates (Figure S10). To the best of our knowledge, this is an example of *Hj*‐aggregates in the BODIPY system. Moreover, potential energy surface scans indicate that the equilibrium intermolecular distance between two Npy‐BDP molecules in an ideal *H*‐aggregate is approximately 3 Å (Figure 2f). However, reduced density gradient analysis reveals that significant Coulombic repulsion associated with π‐π interactions between the phenyl rings induces an interlayer slip of the molecules, leading to the formation of an *Hj‐*type aggregate (shown as the red region in Figure 2g).^[41]^ Consistently, TDA‐TDDFT calculations at the *ω*B97X‐D3/6‐311+G (d, p) level show that, during the structural transition from *H*‐ to *Hj‐*aggregation (Figure 2h,i), the vertical excitation energy of the S_1_ state decreases from 2.15 to 1.96 eV (Table S3). Simultaneously, a new absorption band emerges near 700 nm (highlighted in red line in Figure 2i), in good agreement with the experimental measurements (Figure 2d,e).

Based on the absorption property of Npy‐BDP NPs in the NIR region, 690 nm NIR light source was selected to systematically evaluate the photothermal performance (Figure 3c–f). Under a laser power density of 0.8 W/cm^2^, an 80 μM solution of Npy‐BDP NPs exhibited a significant temperature increase from 27 to 39.5°C (Δ*T* = 12.5°C) within 5 min, demonstrating its photothermal conversion capability (Figure 3c). Further studies revealed that under the same power density, the temperature rise of the Npy‐BDP NPs solutions showed concentration dependence in the range of 20–80 μM, with higher concentrations causing greater temperature increases (Figure 3c). When the concentration was fixed at 80 μM and the laser power density was varied (0.2–0.8 W/cm^2^), the system temperature rose significantly with increasing power density (Figure 3d), indicating a tunable photothermal response. These results collectively confirm that the temperature change of Npy‐BDP NPs solutions is positively correlated with both dye concentration and laser power, highlighting their controllable photothermal conversion properties. Moreover, after five heating‐cooling cycles, the temperature change curves showed no obvious decay (Figure 3e), confirming that Npy‐BDP NPs exhibit no significant photodegradation during the photothermal process and possess excellent photothermal stability. Based on the linear relationship between time and −Ln*θ* during the cooling phase, the photothermal conversion efficiency (*η*) of Npy‐BDP NPs was calculated to be as high as 50% (Figure 3f), verifying its potential for efficient photothermal conversion performance.

**FIGURE 4:**
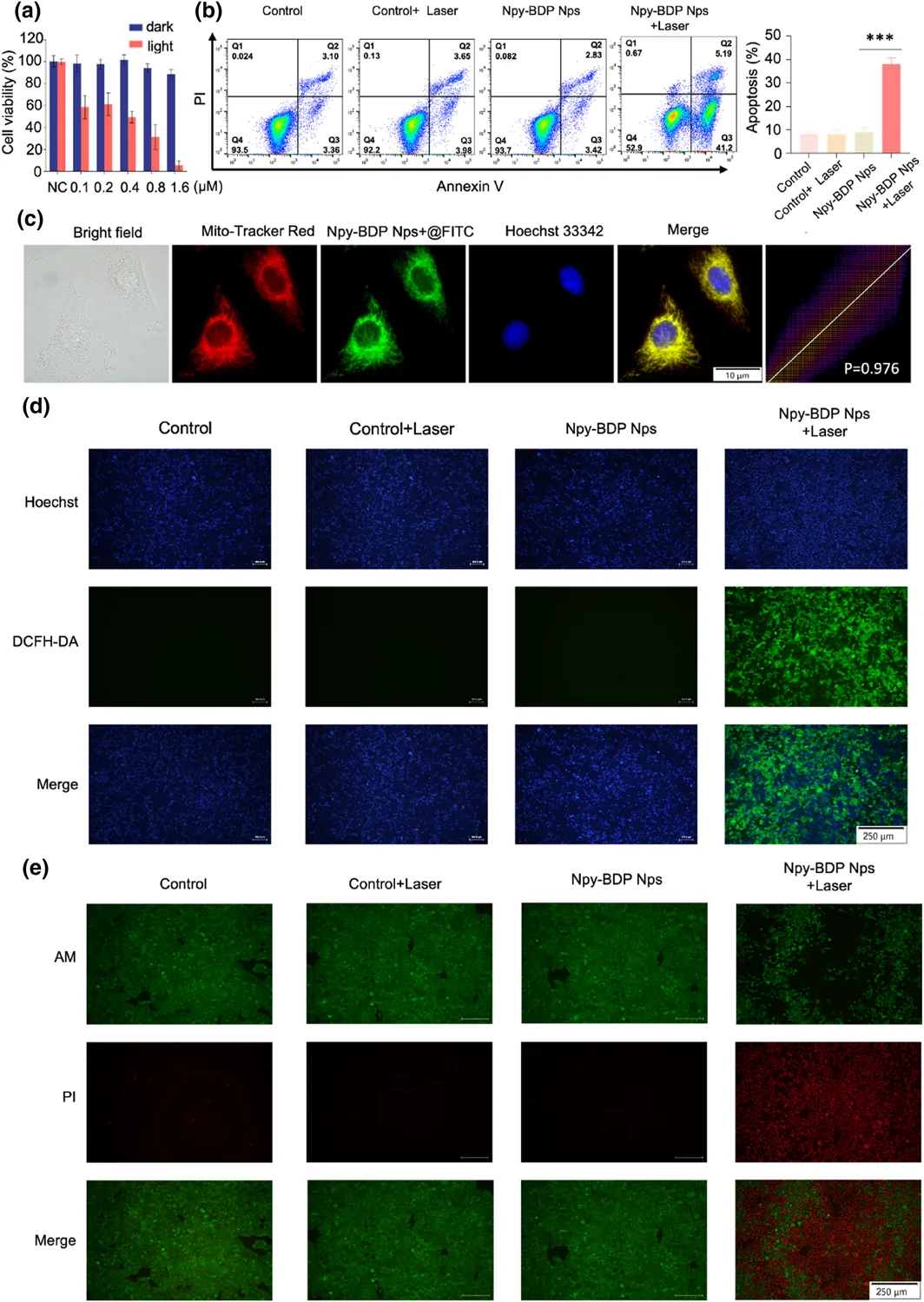
Phototherapeutic efficacy and mechanism of Npy‐BDP NPs in HeLa cells. (a) Cell viability determined by MTT assay under dark conditions or after 690 nm laser irradiation at different concentrations. (b) Flow cytometry analysis of apoptosis and necrosis using Annexin V‐FITC/PI staining for cells treated with different regimens: Control, Laser only, Npy‐BDP NPs only, and Npy‐BDP NPs + Laser. (c) Mitochondrial colocalization of Npy‐BDP NPs in HeLa cells. Scale bars: 10 μm. (d) Intracellular ROS detection using a fluorescent ROS probe under the same irradiation conditions. Scale bars: 250 μm. (e) Live/dead cell staining with calcein‐AM (green, live cells) and PI (red, dead cells) after 690 nm laser irradiation (0.3 W/cm^2^, 10 min). Scale bars: 250 μm. PI, propidium iodide. MTT, 3‐(4,5‐dimethylthiazol‐2‐yl)‐2,5‐diphenyltetrazolium bromide; ROS, reactive oxygen species.

Uptake of Npy‐BDP NPs by HeLa cells increased over time, plateauing at 6 h, which was therefore chosen for later phototoxicity and mechanistic studies (Figure S11). To further evaluate the phototherapeutic activity and cytotoxicity of Npy‐BDP NPs, an 3‐(4,5‐dimethylthiazol‐2‐yl)‐2,5‐diphenyltetrazolium bromide assay was first employed to assess the killing effect on HeLa cells with or without laser irradiation. As shown in Figure 4a, the cell viability of the group irradiated with 690 nm NIR laser was significantly lower than that of the non‐irradiated group at the same concentration. Notably, at a low concentration (0.1 μM), the dark group showed no significant toxicity, whereas the laser‐irradiated group exhibited a substantial cytotoxic effect. These results indicate that Npy‐BDP NPs possess good biosafety and excellent photo‐activated cell‐killing capability. Moreover, the phototherapeutic efficacy was positively correlated with the concentration of the phototherapeutic agent, demonstrating high controllability in therapeutic performance. To further investigate the specific mechanisms and stages of cell death, the Annexin V‐FITC/propidium iodide (PI) double staining was performed combined with flow cytometry to analyze apoptosis.^[42]^ As shown in Figure 4b, the Npy‐BDP NPs plus laser treatment group exhibited an apoptosis rate exceeding 37%, with the majority of cells in the early apoptotic stage, indicating that the cell death induced by phototherapy is predominantly apoptotic. This result suggests that Npy‐BDP NPs not only cause necrosis through direct thermal ablation or membrane damage but also induce programmed cell death by modulating cellular signaling pathways, showing their potential advantage in precisely regulating tumor cell death. The high Pearson's colocalization coefficient (0.976) confirmed efficient mitochondrial targeting of Npy‐BDP NPs (Figure 4c), consistent with a mitochondria‐mediated apoptotic mechanism. Furthermore, to evaluate the intracellular ROS generation capability of Npy‐BDP NPs, a ROS detection kit was used for fluorescence imaging analysis (Figure 4d). The results showed strong green fluorescence signals in the Npy‐BDP NPs plus laser group, which were significantly more intense than other control groups. This indicates that Npy‐BDP NPs can efficiently generate ROS within cells under laser excitation, thereby enhancing oxidative stress and promoting the activation of apoptotic pathways. For direct visual verification of the photo‐induced cell‐killing capability, cells were co‐stained with calcein‐AM (labeling live cells, green fluorescence) and PI (labeling dead cells, red fluorescence).^[43]^ As shown in Figure 4e, the Npy‐BDP NPs plus laser treatment group displayed significantly enhanced red fluorescence compared to the control group, the sole nanoparticle group, and the sole laser group, which provides a clear manifestation that Npy‐BDP NPs can effectively induce tumor cell death under light irradiation. Hence, Npy‐BDP NPs exhibited significant photo‐activated killing effects, clear apoptosis‐inducing ability, and efficient intracellular ROS generation at the cellular level.

**FIGURE 5:**
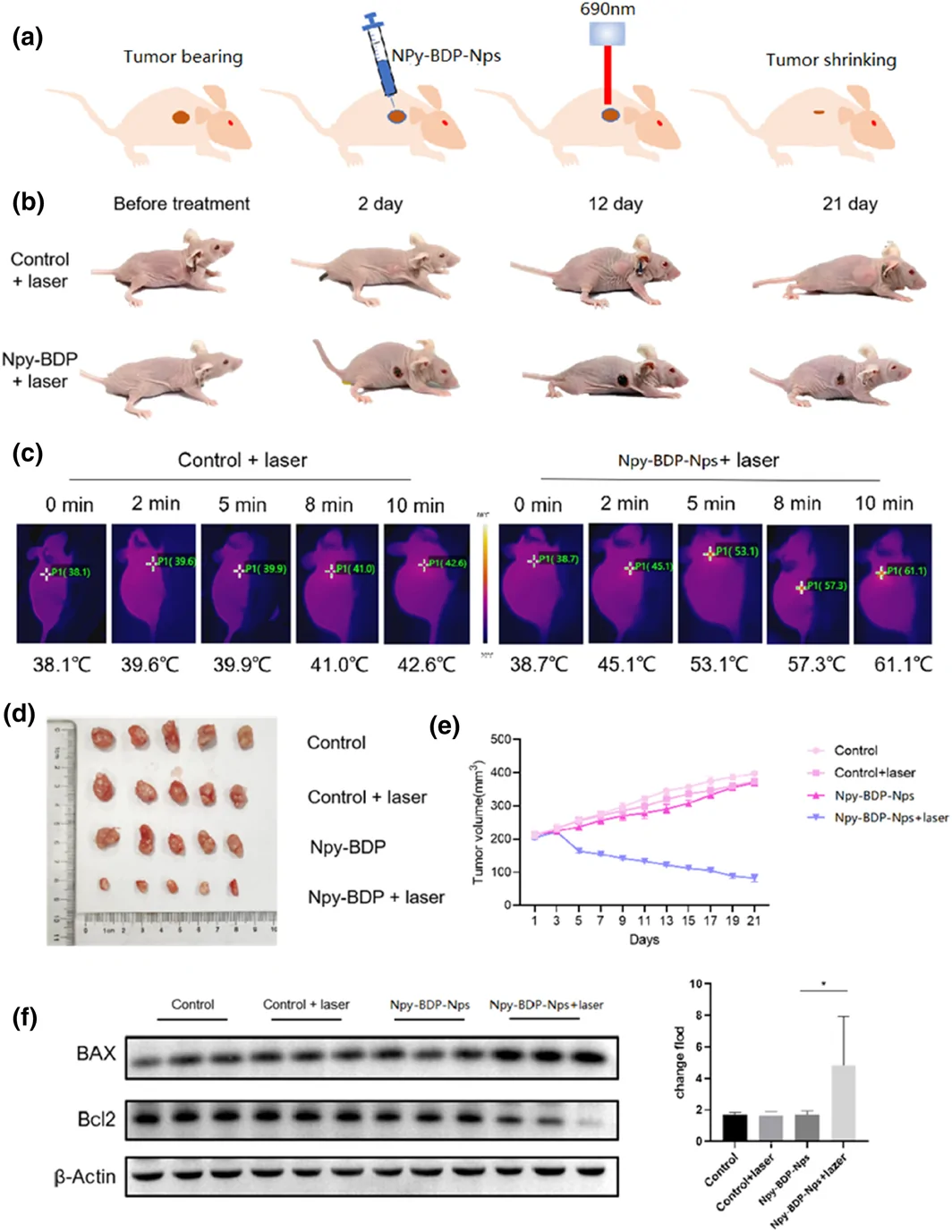
In vivo antitumor efficacy and mechanistic study in a HeLa xenograft mouse model. (a) Schematic illustration of the experimental design. (b) Representative photographs of mice from the control and Npy‐BDP NPs + laser groups at different time points post‐treatment. (c) Infrared thermal images of tumor regions before and after 10 min of 690 nm laser irradiation (0.3 W/cm^2^). (d) Excised tumors from the four treatment groups after 21 days. (e) Statistical graph of changes in tumor size in nude mice in four different treatment groups. (f) Western blot analysis of apoptosis‐related proteins (Bcl‐2 and BAX) in tumor tissues after different treatments.

To systematically evaluate the phototherapeutic efficacy of Npy‐BDP NPs in vivo, a HeLa cell xenograft tumor model was established in mice and designed four experimental groups, control (PBS), sole laser, sole Npy‐BDP NPs, and Npy‐BDP NPs + laser combination therapy. After intratumoral injection of Npy‐BDP NPs (3.5 mg/kg), the tumor site was irradiated with a 690 nm laser (0.3 W/cm^2^) for 10 min, followed by tumor extraction for phototherapeutic assessment (Figure 5a).^[44]^ During treatment, temperature changes in the tumor region were real‐time monitored using infrared thermal imaging. The results showed that the tumor temperature in the Npy‐BDP NPs + laser group rapidly increased under laser irradiation, reaching a maximum of 61.1°C, which was significantly higher than that in the control + laser group (Figure 5c). This demonstrates that Npy‐BDP NPs retain high photothermal conversion efficiency in vivo, enabling precise and localized hyperthermia. Post‐treatment tumor volume was dynamically monitored over 21 days (Figure 5b). The Npy‐BDP NPs + laser group exhibited sustained tumor shrinkage after a single treatment, with no significant recurrence observed throughout the monitoring period, indicating the long‐term tumor growth inhibitory capability. The tumor volume in the Npy‐BDP NPs + laser group was significantly smaller than those in the other groups, providing direct visual evidence of the potent photo‐activated antitumor effect of this molecule in vivo (Figure 5d,e). To further investigate the molecular mechanism underlying Npy‐BDP NPs‐induced tumor cell death under phototherapy, the expression of mitochondrial apoptosis pathway‐related proteins was analyzed by Western blotting (Figure 5f). The results showed that in the Npy‐BDP NPs + laser group, the expression of the pro‐apoptotic protein BAX was significantly upregulated, while the expression of the anti‐apoptotic protein Bcl‐2 was markedly downregulated. These findings suggest that the mitochondrial apoptotic pathway plays a critical role in the phototherapeutic killing mechanism mediated by this system.^[45]^ In addition, H&E staining confirmed no notable organ toxicity, while laser‐treated tumors displayed marked necrosis, demonstrating both biosafety and effective localized antitumor activity (Figure S12). Therefore, Npy‐BDP NPs demonstrated excellent photothermal performance, sustained antitumor efficacy, and clear activation of the mitochondrial apoptotic pathway in vivo, fully highlighting the significant advantages and application potential as a phototherapeutic agent for cancer treatment.

## 3 CONCLUSION

In summary, this work presents a novel quinoline‐fused cyclic pyrrole Npy scaffold that enabled the construction of a symmetrical BODIPY‐based photosensitizer Npy‐BDP. Through the incorporation of heavy‐atom‐free nitrogen atoms and a rigid twisted design, Npy‐BDP simultaneously enhanced ISC and non‐radiative decay, achieving efficient ROS generation and photothermal conversion under 690 nm laser irradiation. Upon nanoparticle encapsulation, Npy‐BDP formed *Hj*‐aggregates in the BODIPY system for the first time, exhibiting dual absorption peaks at 562 and 703 nm and a photothermal conversion efficiency of 53%. Moreover, Npy‐BDP NPs efficiently induced apoptotic cell death in vitro and achieved long‐term tumor suppression in vivo via modulation of BAX/Bcl‐2, demonstrating good biocompatibility and controllable therapeutic efficacy. By innovating the core pyrrole scaffold, this study provides a clear molecular design paradigm that overcomes the limitations of traditional phototherapeutic agents. The development of *Hj*‐aggregating BODIPY nanoparticles establishes a theoretical and experimental foundation for smart molecules with promising potential for clinical translation.

## CONFLICT OF INTEREST STATEMENT

The authors declare no conflicts of interest.

## ETHICS STATEMENT

This experiment was conducted in the compliance with the ethical regulations for animal testing and received approval from the China Medical University Animal Care and Use Committee (No. CMU20241378).

## Supporting information

Supporting Information S1

## Data Availability

The data that supports the findings of this study are available in the supplementary material of this article.
