# Supplementary material for: Hj‐aggregates of quinoline‐fused boron‐dipyrromethene nanoparticles for near‐infrared dualmodal photodynamic and photothermal therapy
Source: Smart Mol. 2026 Aug 23:e70097. Online ahead of print. doi: 10.1002/smo2.70097 (PMC13501090; doi:10.1002/smo2.70097)
Supplement: Supplementary file 1 [file SMO2-9999-0-s001.docx]

**Hj-Aggregates of quinoline-fused BODIPY nanoparticles for NIR dualmodal photodynamic and photothermal therapy**

Bo Zhang, ^‡a^ Chuang Du, ^‡b,c^ Yuewen Yang,^a^ Dongping Xu,^a^ Xin-Dong Jiang,*^a^ Hui-Xin Liu*^b^ and Gaowu Qin^a^

^a^Institute for Strategic Materials and Components, Shenyang University of Chemical Technology, Shenyang, China. Email: xdjiang@syuct.edu.cn

^b^Institute of Life Sciences, China Medical University, Shenyang, China. Email: [liuhx@cmu.edu.cn](mailto:liuhx@cmu.edu.cn)

^c^Department of Gastroenterology, Shengjing Hospital of China Medical University, Shenyang, China

^‡^ These authors contributed equally to this work.

1. [Experimental section](#_Toc133914130)

2. Synthesis

[3. NMR](#_Toc133914131)

[4. HRMS](#_Toc133914132)

[5. Tables](#_Toc133914133) and [Figures](#_Toc133914134)

[6. X-ray data](#_Toc133914135)

7. Reference

***1 Experimental section***

***1.1 General***

Unless otherwise noted, all chemical reagents and organic solvents were of analytical quality and obtained from Energy Chemical & Technology (Shanghai) Co. Ltd. without extra purification. A VARIAN Mercury 500 MHz spectrometer has been employed to record ^1^H NMR spectra. Determined ^1^H NMR chemical shifts (δ) from Me_4_Si are presented in ppm downfield by trace amounts of chloroform (δ = 7.26 ppm). On a VARIAN Mercury 125 MHz spectrometer, ^13^C NMR spectra were obtained in CDCl_3_, reporting in ppm with the internal chloroform signal at δ = 77.0 ppm as the reference. The precise molecular weight of the product was determined using a high-resolution mass spectrometer (HRMS). The absorption and fluorescence spectra were acquired at 25 °C on a UV-2550 spectrophotometer and F-128 fluorescence spectrometer, respectively. A temperature-measuring camera maintains record of the solution temperatures. A fiber connected laser system was implemented to control a 690 nm laser (Changchun New Industries Optoelectronics Technology), which was deployed as the light source for the irradiation experiments. CEL-NP 2000 power meter (Beijing Zhong Jiao Jin Yuan Technology Co, Ltd) was used to quantify the optical power density. The MTT assay was done one a BioTek Synergy H1 microplate reader. To estimate fluorescence visualization, a Confocal Laser Fluorescence Microscope FV1200 (Olympus, Japan) was employed.

***1.2 Computational Methods***

All calculations were carried out using the ORCA.6.1.1 program package.^1-2^ The intermolecular distance of **Npy-BDP** in the H-aggregated configuration was determined via a potential energy scan at the *ω*B97X-D3/6-311+G (d, p) level.^3,4^ Excited-state energies and absorption spectra were evaluated by computing 80 singlet excited states using TDA-*ω*B97X-D3/6-311+G (d, p).^5,6^ The lowest ten excited states are listed in Table S3. Reduced density gradient (RDG) analysis was carried out to characterize the π-π interactions between the phenyl rings in the *H*-aggregate.

***1.3 Singlet oxygen assay***

The ^1^O_2_ production in toluene was determined utilizing 1,3-diphenylisobenzofuran (DPBF) as a ^1^O_2_ trap. The absorbance of DPBF at 418 nm was adjusted to about 1.5 in toluene and the absorbance of the dyes was adjusted to about 0.8 at 690 nm. The 690 nm laser light was used for the excitation of the dye, the optical radiation power was 0.1 W/cm^2^, and the illumination time was 10 min. Methylene blue (MB) was used as a reference (Φ_Δ_ = 57% in DCM).

The ^1^O_2_ production efficiency of the dyes was calculated from the decrease of absorbance of DPBF (monitored at 418 nm) according to the following equation:

Where “*sam*” represents the dye with unknown ^1^O_2_ quantum yield and “*MB*” represents the reference methylene blue. “*m*” is the slope of DPBF absorption peak decline, “*F*” is the absorption correction factor, F = 1-10^-O.D.^. *O.D.* represents the absorption value of the sample at the wavelength of light radiation.

***1.4 Reactive oxygen species (ROS) investigation***

A 10 μM solution of DCFH in DMSO was mixed with a 10 μM of nanoparticle dispersions of compounds to obtain the mixed solutions. Upon irradiation with a 690 nm laser (0.1 W cm⁻²), the fluorescence of the mixtures was measured after 1 min of exposure, with total irradiation lasting 10 min.

***1.5 Calculation of photothermal efficiency***

The photothermal conversion efficiencies (*η*) was calculated using the following method:

*h* means heat transfer coefficient, *s* was for container surface area, *Q_Dis_* stands for heat dispersed from the laser via the solvent and container, *I* was for laser power, and *A* represents for absorbance at excitation wavelength. *η* denotes photothermal conversion efficiency.

*m* is the total quantity of the photothermal reagent containing solution, *C* represents the temperature coefficient, and *τ*_s_ is the relevant time constant.

The temperature of the driving force is a non-dimensional parameter termed *θ*.

*T* is the current temperature, *T_Max_* is the highest steady state temperature, and *T_surr_* denotes the surrounding temperature.

***1.6 MTT Assay***

The in vitro cytotoxicity of **Npy-BDP** NPs against HeLa cells was evaluated by the MTT assay. Cells were seeded at 5 × 10^3^ per well in 96-well plates and incubated overnight at 37 °C in a humidified 5% CO_2_ atmosphere. After exposure to serial concentrations of Npy-BDP-NPs for 12 h, the cells were irradiated with a 690 nm laser at 0.3 W cm⁻² for 10 min. Following a further 24 h culture, 20 μL MTT solution (5 mg mL^-1^) was added to each well. After 4 h incubation, the supernatant was removed, DMSO was added, and the plates were gently shaken for 10 min before absorbance was measured on a microplate reader to determine cell viability.

***1.7 Calcein-AM/propidium iodide (PI) staining assay***

The cells were categorized into four distinct groups in the following experiments: the control group received no treatment; the light group underwent 690 nm laser irradiation treatment; the NPs group was treated with **Npy-BDP** NPs (1.6 μM); and the NPs (1.6 μM) with 690 nm laser irradiation of last group (Npy-BDP-NPs plus light group) received combined treatment of NPs and laser irradiation. The calcein-AM /PI staining kit (Wuhan Servicebio Technology Co., Ltd.) was employed to assess the cellular death rate. To assess the phototherapeutic efficacy of **Npy-BDP** NPs, HeLa cells were incubated with the compound and then irradiated with a 690 nm laser for 10 min. After irradiation, the cells were co-stained with calcein-AM (live indicator, green) and propidium iodide (dead indicator, red) following the kit instructions. Briefly, HeLa cells were seeded into 24-well plates at 2 × 10^4^ cells per well, treated with 4 μmol/L **Npy-BDP** NPs for 12 h, and exposed to 690 nm irradiation at 0.3 W/cm^2^ for 10 min.

To evaluate the antitumor efficacy of **Npy-BDP** NPs, HeLa cells were loaded with 4 μmol/L **Npy-BDP** NPs for 12 h, irradiated with a 690 nm laser at 0.3 W/cm^2^ for 10 min, and immediately stained with DCFH-DA fluorescent probe according to the kit instructions. Cells were pre-seeded in 24-well plates at 2 × 10^4^ cells per well.

***1.8 Flow cytometry analysis of apoptosis***

Flow cytometry was employed to quantify apoptosis in HeLa cells after **Npy-BDP**-NPs-mediated photothermal therapy. Cells were stained with Annexin V-FITC/PI Apoptosis Detection Kit (YEASEN, 40302ES20, China) following the manufacturer's protocol and analysed on a BD FACSCelesta flow cytometer. The sum of early- and late-apoptotic populations was taken as the total apoptotic rate (n = 3 per group).

***1.9 Tumor models***

For in vivo studies, 2 × 10^6^ HeLa cells in 100 μL PBS were subcutaneously inoculated into the forelimb of female BALB/c nude mice (5–6 weeks, 17–19 g) to establish a cervical carcinoma xenograft model. All procedures conformed to institutional animal-use guidelines and were approved by the China Medical University Animal Care and Use Committee (approval No. CMU20241378).

***1.10 In vivo antitumor efficacy***

One week after tumour inoculation, the mice were randomly assigned to four groups (n = 5): control, control+laser, **Npy-BDP** NPs, and **Npy-BDP** NPs+laser. Mice in the **Npy-BDP** NPs groups received an intratumoural injection of the agent. And 24 h later the laser-treated groups were irradiated with a 690 nm laser for 10 min at 0.3 W/cm^2^, whereas control animals received an equal volume of PBS. Intratumoural temperature changes were monitored in real time with an infrared thermal camera. Tumour volumes were measured every 2 days. All mice were sacrificed on day 21, or earlier if tumour size exceeded 2000 mm^3^. Mitochondrial apoptosis-related proteins in the resected tumors were analysed by Western blot to assess treatment-induced cell death.

***2 Synthesis***

***2.1 Synthesis of the pyrrole Npy***

Under N₂, LDA (0.4 mL, 3.18 mmol) in THF (30 mL) was added to 3,4-dihydroacridin-1(2H)-one (627 mg, 3.18 mmol) at 0 °C. The mixture was cooled to −78 °C for 15 min, followed by addition of 3-phenyl-2H-azirine (0.6 mL, 5.48 mmol) and stirred for 10 min. The reaction was then allowed to warm to room temperature and stirred for an additional 2.5 h. The mixture was extracted with CH₂Cl₂ (3 × 80 mL). The combined organic layers were washed with brine (3 × 80 mL), dried over anhydrous Na₂SO₄, and concentrated under reduced pressure. The crude product was purified by column chromatography on silica gel (*n*-hexane/CH₂Cl₂ = 1:2) to afford **Npy** as a brown solid (508 mg, 54%). ^1^H NMR (400 MHz, CDCl_3_): δ (ppm) 9.24 (s, 1H), 7.89 (d, ^3^J = 8.0 Hz, 1H), 7.67 (s, 1H), 7.49 (d, ^3^J = 8.0 Hz, 1H), 7.44 (t, ^3^J = 8.0 Hz, 1H), 7.36 (d, ^3^J = 8.0 Hz, 2H), 7.30 (t, ^3^J = 8.0 Hz, 3H), 7.16 (t, ^3^J = 8.0 Hz, 1H), 6.97 (d, ^3^J = 2.4 Hz, 1H), 3.23 (d, ^3^J = 7.2 Hz, 2H), 3.02 (d, ^3^J = 7.2 Hz, 2H). ^13^C NMR (125 MHz, CDCl_3_): δ 158.7, 145.5, 135.4, 128.7, 128.3, 128.2, 127.8, 127.3, 127.2, 127.1, 126.4, 126.0, 124.3, 123.3, 122.4, 119.9, 118.4, 29.8, 20.9.

***2.2 Synthesis of Npy-BDP***

Under N₂, pyrrole **Npy** (300 mg, 1 mmol) was condensed with *p*-toluenesulfonic acid (87.2 mg, 0.51 mmol) and triethyl orthoformate (5 mL) at room temperature for 4 h. The reaction mixture was then concentrated under reduced pressure, and the residue was recrystallized from n-hexane. The resulting solid was dissolved in CH₂Cl₂ (30 mL), followed by the addition of triethylamine (0.4 mL). After stirring for 15 min, BF₃·Et₂O (0.6 mL, 4.86 mmol) was slowly added dropwise. The mixture was then heated in an oil bath at 80 °C for 2.5 h. After completion, the reaction mixture was extracted with CH₂Cl₂, and the combined organic layers were evaporated. The crude product was purified by column chromatography on silica gel (CH₂Cl₂) to afford **Npy-BDP** as a green solid (115.94 mg, 35.2%). ^1^H NMR (400 MHz, CDCl_3_): δ (ppm) 8.67-8.71 (m, 2H), 8.34 (d, ^3^J = 8.0 Hz, 2H), 8.28 (d, ^3^J = 8.0 Hz, 2H), 7.91-7.93 (m, 1H), 7.79 (t, ^3^J = 8.0 Hz, 2H), 7.58-7.61 (m, 4H), 7.51-7.55 (m, 6H), 7.14-7.19 (m, 2H), 3.29 (d, ^3^J = 7.2 Hz, 4H), 2.85 (d, ^3^J = 7.2 Hz, 4H). ^13^C NMR (125 MHz, CDCl_3_): δ 168.3, 160.9, 148.3, 140.6, 137.3, 1335, 132.5,131.8,131.7, 131.5, 130.9, 130.2, 129.8, 129.6,129.5, 129.4, 128.4, 127.5, 29.5, 20.7. HRMS m/z calcd for C_43_H_30_BF_2_N_4_^+^ (M+H)^+^ 651.25261, found: 651.25287.

***3 NMR***

**Figure S1.** ^1^H NMR spectrum (400 MHz, CDCl_3_) of pyrrole **Npy**.

**Figure S2.** ^13^C NMR spectrum (400 MHz, CDCl_3_) of pyrrole **Npy**.

**Figure S3.** ^1^H NMR spectrum (400 MHz, CDCl_3_) of **Npy-BDP**.

**Figure S4.** ^13^C NMR spectrum (400 MHz, CDCl_3_) of **Npy-BDP**.

***4 HRMS***

HRMS m/z calcd for C_43_H_30_BF_2_N_4_^+^ (M+H)^+^ 651.25261, found: 651.25287.

**Figure S5.** High-resolution mass spectrometry of **Npy-BDP**.

**5 Tables and Figures**

**Table S1** Comparison of photophysical properties of BODIPYs in CH_2_Cl_2_ at 298 K.

| Dye | Structure | λ_abs_/λ_em_ [nm] | ε  [M^-1^ cm^-1^] | *Φ*_f_ | *Φ*_Δ_ | η | Reference |
| --- | --- | --- | --- | --- | --- | --- | --- |
| BDP-2b |  | 538/560 | 71418 | 0.537 |  |  | *New J. Chem.* ***2025****, 49 (26): 11326–11333.* |
| carbonyl-BDP |  | 546/558 | 85000 | 0.13 | 0.68 | / | *Synlett,* ***2024****, 35(01): 91-94* |
| 4PhDPM |  | 564/593 | 79000 | 0.92 | */* | */* | *Spectrochim Acta A Mol Biomol Spectrosc.* ***2014****, 117, 323-329.* |
| DMA-BOD |  | 562/577 | 96990 | 0.025 | 0.04 | */* | *Chem. Sci.,* ***2020****, 11 (25): 6479–6484.* |
| BDP-1 |  | 564/572 | 121000 | 0.31 | 0.55 | / | *Organic Letters,* ***2020****, 22 (14), 5535-5539* |
| H-B |  | 588/598 | 191000 | 0.818 |  |  | *Org. Chem. Front.* ***2026****, 13 (1): 13–24.* |
| helical-BDP |  | 630/649 | 176000 | 0.21 | 0.36 | / | *Angew Chem Int Ed Engl.* ***2020****,59(37):16114-16121.* |
| BODIPY 8 |  | 632/646 | 123000 | 0.82 | */* | */* | *Tetrahedron Lett.* ***2018****, 59, 546–549.* |
| BODIPY 7 |  | 648/659 | 130000 | 0.65 | */* | */* | *Tetrahedron Lett.* ***2018****, 59, 546–549.* |
| BODIPY B-4b |  | 628/648 | 95000 | 0.22 | 0.41 | */* | *Chem. Commun.,* ***2023****, 59, 12330–12333* |
| Npy-BDP |  | 660/676 |  |  |  |  | ***This work*** |

**Table S2** Photophysical properties of **Npy-BDP** in various solvents at 298 K

| Solvent | λ_abs_/λ_em_ (nm) | Stokes’ shift (nm) | Fwhm (nm) |
| --- | --- | --- | --- |
| DCM | 660/676 | 16 | 24 |
| DMF | 658/677 | 19 | 27 |
| DMSO | 662/681 | 18 | 27 |
| THF | 658/674 | 16 | 25 |
| Toluene | 664/681 | 17 | 24 |
| CHCl_3_ | 660/677 | 17 | 24 |

**Table S3** Vertical excitation energies (E_VE_, in eV) in the first 10 excited states with their oscillator strengths *(f)* of *H-aggregates* and *Hj*-aggregates calculated by the TDA-*ω*B97X-D3/6-311+G (d, p) method.

| **Excited States** | ***H-aggregates*** | | ***Hj*-aggregates** | |
| --- | --- | --- | --- | --- |
|  | E_VE_ | *f* | E_VE_ | *f* |
| **S_1_** | 2.15 | 0.00029 | 1.96 | 0.00013 |
| **S_2_** | 2.38 | 1.34210 | 2.37 | 0.20108 |
| **S_3_** | 2.66 | 0.01100 | 2.45 | 1.32776 |
| **S_4_** | 2.73 | 0.60546 | 2.65 | 0.00835 |
| **S_5_** | 2.83 | 0.00805 | 2.76 | 0.01119 |
| **S_6_** | 2.89 | 0.40889 | 2.87 | 0.01119 |
| **S_7_** | 3.10 | 0.00002 | 2.90 | 0.23214 |
| **S_8_** | 3.15 | 0.00010 | 3.08 | 0.38009 |
| **S_9_** | 3.65 | 0.00039 | 3.38 | 0.00166 |
| **S_10_** | 3.67 | 0.02425 | 3.43 | 0.03353 |

**Figure S6.** Normalized spectra (up) and Emission spectra (down) of **Npy-BDP** at various solvents at 298 K.

**Figure S7.** Time-dependent photodegradation of DPBF with (a) MB and (b) **Npy-BDP** in DMSO (690 nm laser, 0.3 W/cm²); (c) The degradation rate curve of DPBF corresponding to Npy-BDP (in red) and MB (in black).

**Figure S8.** (a) Time-dependent photodegradation of ABDA with **Npy-BDP** NPs in PBS under 690 nm laser (0.1 W/cm²); (b) The degradation rate curve of DPBF corresponding to **Npy-BDP** (in red) and the degradation rate curve of ABDA corresponding to **Npy-BDP** NPs (in purple); (c) ROS generation of **Npy-BDP** NPs (10 μM) detected by DHR123 (10 μM) in PBS under 690 nm laser (0.1 W/cm², 10 min); (d) The SOD quenching assay was used to verify the specificity of the DHR123 (10 μM) probe for superoxide anion (0.1 W/cm², 10 min).

**Figure S9.** Particle size and Zeta potential diagrams of **Npy-BDP** NPs aqueous solution over 0-7 days

**Figure S10.** (a) Time-resolved emission spectra (TRES) of compound **Npy‑BDP** recorded at delay times from 17.3 to 80 ns upon excitation at 456 nm, each spectrum was integrated over a time gate of 1.3 ns. (b) The corresponding 2D pseudo-color time-resolved emission map, where the color scale represents the emission intensity in counts (logarithmic scale). All measurements were performed in THF/H₂O (v/v = 1:9) at a concentration of 0.8 mM at 298 K.

**Figure S11.** Cell uptake of **Npy-BDP** NPs by HeLa cells. HeLa cells were incubated with **Npy-BDP** NPs at a concentration of 20 μM for 0, 1, 2, 4, 6, and 8 hours. After incubation, the cell uptake efficiency was evaluated using a fluorescence microscope.

**Figure S12.** H&E staining of major organs and tumor of Control + laser group and **Npy-BDP** + laser group. Scale: 100 μm.

**6. Xray data for Npy**

| **Table 1 Crystal data and structure refinement for Npy.** | |
| --- | --- |
| Identification code | **Npy** |
| Empirical formula | C_21_H_16_N_2_ |
| Formula weight | 296.36 |
| Temperature/K | 100.00(10) |
| Crystal system | monoclinic |
| Space group | P2_1_/n |
| a/Å | 17.6813(4) |
| b/Å | 9.50385(17) |
| c/Å | 20.0897(5) |
| α/° | 90 |
| β/° | 105.246(3) |
| γ/° | 90 |
| Volume/Å^3^ | 3257.06(13) |
| Z | 8 |
| ρ_calc_g/cm^3^ | 1.209 |
| μ/mm^‑1^ | 0.551 |
| F(000) | 1248.0 |
| Crystal size/mm^3^ | 0.14 × 0.12 × 0.11 |
| Radiation | Cu Kα (λ = 1.54184) |
| 2Θ range for data collection/ | 5.934 to 147.056 |
| Index ranges | -21 ≤ h ≤ 21, -11 ≤ k ≤ 9, -24 ≤ l ≤ 24 |
| Reflections collected | 26259 |
| Independent reflections | 6437 [R_int_ = 0.0570, R_sigma_ = 0.0492] |
| Data/restraints/parameters | 6437/0/415 |
| Goodness-of-fit on F^2^ | 1.050 |
| Final R indexes [I>=2σ (I)] | R_1_ = 0.0468, wR_2_ = 0.1194 |
| Final R indexes [all data] | R_1_ = 0.0565, wR_2_ = 0.1269 |
| Largest diff. peak/hole / e Å^-3^ | 0.18/-0.23 |

**Crystal structure determination of [Npy]**

**Crystal Data** for C_21_H_16_N_2_ (*M* =296.36 g/mol): monoclinic, space group P2_1_/n (no. 14), *a* = 17.6813(4) Å, *b* = 9.50385(17)Å, *c* = 20.0897(5)Å, *β* = 105.246(3), *V*= 3257.06(13)Å^3^, *Z* = 8, *T* = 100.00(10) K, μ(Cu Kα)= 0.551 mm^-1^, *Dcalc* = 1.209 g/cm^3^, 26259 reflections measured (5.934 ≤ 2Θ ≤ 147.056°), 6437 unique (*R*_int_ = 0.0570, R_sigma_ = 0.0492) which were used in all calculations. The final *R*_1_ was 0.0468 (I > 2σ(I)) and *wR*_2_ was 0.1269 (all data).

**Refinement model description**

| **Table 2 Fractional Atomic Coordinates (×10^4^) and Equivalent Isotropic Displacement Parameters (Å^2^×10^3^) for Npy. U_eq_ is defined as 1/3 of the trace of the orthogonalised U_IJ_ tensor.** | | | | |
| --- | --- | --- | --- | --- |
| **Atom** | ***x*** | ***y*** | ***z*** | **U(eq)** |
| N1 | 5784.0(8) | 1825.1(14) | 5737.3(7) | 33.6(3) |
| N2 | 5789.9(8) | 3930.4(14) | 3505.7(7) | 33.3(3) |
| C1 | 3714.9(10) | 2030.1(17) | 5739.6(9) | 32.8(3) |
| C2 | 3206.4(10) | 3180.9(19) | 5594.0(9) | 36.9(4) |
| C3 | 2494.5(11) | 3160(2) | 5766.5(10) | 42.9(4) |
| C4 | 2278.5(11) | 1984(2) | 6077.7(11) | 47.0(5) |
| C5 | 2770.1(12) | 833(2) | 6218.9(11) | 46.0(5) |
| C6 | 3484.2(11) | 851.4(19) | 6053.7(10) | 39.1(4) |
| C7 | 5183.2(10) | 1618.6(18) | 6036.0(9) | 34.9(4) |
| C8 | 4488.7(9) | 2063.3(16) | 5590.1(9) | 31.5(3) |
| C9 | 4685.7(9) | 2576.9(16) | 4993.0(9) | 30.3(3) |
| C10 | 4198.8(10) | 3085.6(19) | 4307.5(9) | 35.8(4) |
| C11 | 4672.6(10) | 4090.3(18) | 3986.2(9) | 34.3(4) |
| C12 | 5485.7(9) | 3575.3(16) | 4012.9(9) | 30.8(3) |
| C13 | 5919.1(9) | 2810.6(16) | 4608.8(9) | 29.8(3) |
| C14 | 5488.2(9) | 2422.8(16) | 5102.2(9) | 30.1(3) |
| C15 | 6698.2(9) | 2527.2(17) | 4669.0(9) | 32.1(3) |
| C16 | 7042.8(9) | 2941.5(17) | 4142.3(9) | 33.1(3) |
| C17 | 6560.7(9) | 3618.3(18) | 3551.9(9) | 33.6(4) |
| C18 | 6883.3(11) | 4015(2) | 3007.0(10) | 40.6(4) |
| C19 | 7659.5(11) | 3773(2) | 3053.2(10) | 45.8(4) |
| C20 | 8142.9(11) | 3122(2) | 3641.6(10) | 43.6(4) |
| C21 | 7842.3(10) | 2707.7(19) | 4173.1(10) | 39.4(4) |
| N3 | 4976.0(8) | 5789.7(14) | 2420.1(7) | 32.5(3) |
| N4 | 2500.4(8) | 3350.2(14) | 1395.5(7) | 31.3(3) |
| C22 | 2755.0(9) | 2089.9(16) | 1711.8(8) | 29.9(3) |
| C23 | 2232.3(10) | 926.2(17) | 1616.8(9) | 35.1(4) |
| C24 | 2466.3(10) | -329.8(17) | 1936.9(9) | 35.3(4) |
| C25 | 3233.3(10) | -506.0(17) | 2363.0(9) | 33.5(4) |
| C26 | 3747.4(10) | 597.1(17) | 2461.6(9) | 33.4(4) |
| C27 | 3524.7(9) | 1909.6(16) | 2137.5(9) | 29.6(3) |
| C28 | 4038.9(9) | 3070.4(16) | 2222.7(9) | 31.3(3) |
| C29 | 3781.8(9) | 4330.9(16) | 1917.9(8) | 28.4(3) |
| C30 | 2985.6(9) | 4430.0(16) | 1501.5(9) | 30.5(3) |
| C31 | 2680.6(10) | 5855.0(17) | 1224.5(10) | 36.4(4) |
| C32 | 3282.3(10) | 6826.7(18) | 1027.7(10) | 36.1(4) |
| C33 | 4055.0(10) | 6779.1(16) | 1569.0(9) | 31.4(3) |
| C34 | 4249.7(9) | 5600.3(16) | 1975.6(9) | 30.4(3) |
| C35 | 5246.4(10) | 7081.7(17) | 2301.9(9) | 34.0(4) |
| C36 | 4700.4(10) | 7737.2(17) | 1773.4(9) | 33.4(3) |
| C37 | 4814.3(10) | 9124.1(18) | 1491.2(10) | 37.6(4) |
| C38 | 5350.9(11) | 10080.3(19) | 1891.1(11) | 42.6(4) |
| C39 | 5488.6(13) | 11373(2) | 1626.7(14) | 56.4(6) |
| C40 | 5092.1(16) | 11749(2) | 968.1(16) | 71.4(7) |
| C41 | 4565.7(15) | 10826(2) | 565.3(15) | 64.7(7) |
| C42 | 4425.1(12) | 9517(2) | 823.4(12) | 47.3(5) |

| **Table 3 Anisotropic Displacement Parameters (Å^2^×10^3^) for Npy. The Anisotropic displacement factor exponent takes the form: -2π^2^[h^2^a*^2^U_11_+2hka*b*U_12_+…].** | | | | | | |
| --- | --- | --- | --- | --- | --- | --- |
| **Atom** | **U_11_** | **U_22_** | **U_33_** | **U_23_** | **U_13_** | **U_12_** |
| N1 | 25.5(7) | 37.9(7) | 36.6(8) | 6.2(6) | 7.0(6) | 1.6(5) |
| N2 | 24.5(7) | 38.3(7) | 35.8(8) | 2.6(6) | 5.7(6) | -2.6(5) |
| C1 | 29.5(8) | 37.1(8) | 31.5(8) | -4.8(7) | 7.6(7) | -6.5(6) |
| C2 | 30.6(9) | 40.7(9) | 39.3(10) | -2.5(7) | 8.9(7) | -2.2(7) |
| C3 | 31.7(9) | 52.3(10) | 44.4(11) | -9.6(8) | 9.3(8) | 0.7(8) |
| C4 | 35.3(10) | 61.7(12) | 49.2(11) | -15.6(9) | 19.9(9) | -11.3(9) |
| C5 | 48.3(11) | 47.4(10) | 49.1(11) | -8.0(8) | 24.6(9) | -16.2(8) |
| C6 | 41.0(10) | 38.3(9) | 41.1(10) | -3.1(7) | 16.3(8) | -6.5(7) |
| C7 | 30.1(8) | 38.4(8) | 36.4(9) | 6.5(7) | 9.4(7) | -0.3(7) |
| C8 | 27.8(8) | 31.0(7) | 35.7(9) | 1.5(6) | 8.2(7) | -2.1(6) |
| C9 | 24.3(7) | 30.8(7) | 34.6(8) | 0.1(6) | 5.7(6) | -3.0(6) |
| C10 | 23.5(8) | 44.9(9) | 38.4(9) | 6.1(7) | 7.2(7) | 0.5(7) |
| C11 | 26.3(8) | 40.4(9) | 35.2(9) | 5.9(7) | 6.0(7) | 2.3(6) |
| C12 | 24.7(8) | 32.2(8) | 34.0(9) | 0.0(6) | 4.9(6) | -3.4(6) |
| C13 | 24.9(8) | 29.8(7) | 33.6(8) | -0.8(6) | 6.0(6) | -1.4(6) |
| C14 | 24.7(8) | 29.9(7) | 34.4(9) | 0.7(6) | 5.7(6) | -1.0(6) |
| C15 | 25.2(8) | 33.2(8) | 36.4(9) | 1.9(7) | 5.4(7) | -0.7(6) |
| C16 | 24.7(8) | 35.0(8) | 39.0(9) | -1.8(7) | 7.4(7) | -2.5(6) |
| C17 | 24.8(8) | 39.2(8) | 36.3(9) | 1.1(7) | 7.1(7) | -3.9(6) |
| C18 | 30.6(9) | 53.3(10) | 37.2(10) | 4.3(8) | 7.6(7) | -4.3(7) |
| C19 | 34.0(10) | 63.2(12) | 43.4(11) | 5.2(9) | 16.1(8) | -3.1(8) |
| C20 | 27.3(9) | 55.6(11) | 49.9(11) | 4.5(9) | 13.6(8) | 1.0(8) |
| C21 | 26.8(8) | 45.8(9) | 45.2(10) | 3.7(8) | 9.0(7) | 1.8(7) |
| N3 | 25.3(7) | 34.5(7) | 36.2(8) | 6.8(6) | 5.3(6) | -2.7(5) |
| N4 | 25.6(7) | 33.2(7) | 33.8(7) | -1.4(5) | 5.8(6) | 0.9(5) |
| C22 | 25.4(8) | 32.4(8) | 32.3(8) | -3.2(6) | 8.2(6) | 0.1(6) |
| C23 | 26.5(8) | 38.1(8) | 39.0(9) | -3.2(7) | 5.7(7) | -2.9(6) |
| C24 | 32.0(9) | 33.7(8) | 41.5(10) | -4.8(7) | 11.8(7) | -6.9(7) |
| C25 | 33.4(9) | 31.5(8) | 38.3(9) | 2.1(7) | 14.2(7) | 1.7(6) |
| C26 | 26.1(8) | 35.3(8) | 39.7(9) | 2.6(7) | 10.2(7) | 0.8(6) |
| C27 | 25.1(8) | 32.3(8) | 32.9(8) | -0.8(6) | 10.1(6) | 0.6(6) |
| C28 | 22.9(7) | 35.7(8) | 35.1(9) | 1.0(7) | 7.1(6) | -0.4(6) |
| C29 | 24.6(8) | 31.9(7) | 29.5(8) | 0.2(6) | 8.4(6) | 0.0(6) |
| C30 | 26.2(8) | 33.2(8) | 32.6(8) | -1.0(6) | 8.4(7) | 0.8(6) |
| C31 | 27.2(8) | 34.6(8) | 43.2(10) | 1.9(7) | 1.9(7) | 1.8(6) |
| C32 | 32.7(9) | 34.1(8) | 39.2(9) | 5.6(7) | 5.8(7) | 2.9(7) |
| C33 | 28.2(8) | 33.0(8) | 34.1(9) | 1.4(6) | 10.0(7) | 1.6(6) |
| C34 | 23.7(7) | 34.0(8) | 34.0(8) | 0.0(7) | 8.3(6) | -0.9(6) |
| C35 | 28.5(8) | 35.7(8) | 39.1(9) | 0.9(7) | 11.4(7) | -5.5(6) |
| C36 | 31.2(8) | 33.2(8) | 38.6(9) | 2.2(7) | 14.1(7) | -0.1(6) |
| C37 | 32.9(9) | 35.3(8) | 49.7(11) | 4.8(7) | 20.0(8) | 2.0(7) |
| C38 | 36.8(9) | 37.1(9) | 59.8(12) | 1.9(8) | 22.8(9) | -2.0(7) |
| C39 | 47.0(12) | 39.5(10) | 89.3(17) | 7.0(10) | 29.5(12) | -4.6(8) |
| C40 | 67.2(16) | 47.0(12) | 103(2) | 30.7(13) | 27.4(15) | -5.3(11) |
| C41 | 58.4(14) | 60.9(13) | 75.1(16) | 34.0(12) | 18.2(12) | 6.0(11) |
| C42 | 41.3(10) | 45.2(10) | 57.1(12) | 15.5(9) | 15.9(9) | 1.9(8) |

| **Table 4 Bond Lengths for Npy.** | | | | | |  |
| --- | --- | --- | --- | --- | --- | --- |
| **Atom** | **Atom** | **Length/Å** |  | **Atom** | **Atom** | **Length/Å** |
| N1 | C7 | 1.365(2) |  | N3 | C34 | 1.370(2) |
| N1 | C14 | 1.369(2) |  | N3 | C35 | 1.361(2) |
| N2 | C12 | 1.315(2) |  | N4 | C22 | 1.375(2) |
| N2 | C17 | 1.374(2) |  | N4 | C30 | 1.318(2) |
| C1 | C2 | 1.397(2) |  | C22 | C23 | 1.422(2) |
| C1 | C6 | 1.398(2) |  | C22 | C27 | 1.414(2) |
| C1 | C8 | 1.476(2) |  | C23 | C24 | 1.367(2) |
| C2 | C3 | 1.391(3) |  | C24 | C25 | 1.411(3) |
| C3 | C4 | 1.383(3) |  | C25 | C26 | 1.367(2) |
| C4 | C5 | 1.380(3) |  | C26 | C27 | 1.414(2) |
| C5 | C6 | 1.387(3) |  | C27 | C28 | 1.411(2) |
| C7 | C8 | 1.382(2) |  | C28 | C29 | 1.368(2) |
| C8 | C9 | 1.421(2) |  | C29 | C30 | 1.439(2) |
| C9 | C10 | 1.499(2) |  | C29 | C34 | 1.450(2) |
| C9 | C14 | 1.385(2) |  | C30 | C31 | 1.509(2) |
| C10 | C11 | 1.521(2) |  | C31 | C32 | 1.537(2) |
| C11 | C12 | 1.506(2) |  | C32 | C33 | 1.507(2) |
| C12 | C13 | 1.437(2) |  | C33 | C34 | 1.376(2) |
| C13 | C14 | 1.448(2) |  | C33 | C36 | 1.433(2) |
| C13 | C15 | 1.377(2) |  | C35 | C36 | 1.381(2) |
| C15 | C16 | 1.409(2) |  | C36 | C37 | 1.469(2) |
| C16 | C17 | 1.420(2) |  | C37 | C38 | 1.404(3) |
| C16 | C21 | 1.416(2) |  | C37 | C42 | 1.388(3) |
| C17 | C18 | 1.412(2) |  | C38 | C39 | 1.385(3) |
| C18 | C19 | 1.370(3) |  | C39 | C40 | 1.372(4) |
| C19 | C20 | 1.407(3) |  | C40 | C41 | 1.377(4) |
| C20 | C21 | 1.370(3) |  | C41 | C42 | 1.395(3) |

| **Table 5 Bond Angles for Npy.** | | | | | | | | |
| --- | --- | --- | --- | --- | --- | --- | --- | --- |
| **Atom** | **Atom** | **Atom** | **Angle/˚** |  | **Atom** | **Atom** | **Atom** | **Angle/˚** |
| C7 | N1 | C14 | 108.59(14) |  | C35 | N3 | C34 | 108.48(14) |
| C12 | N2 | C17 | 119.52(14) |  | C30 | N4 | C22 | 118.90(14) |
| C2 | C1 | C6 | 118.28(16) |  | N4 | C22 | C23 | 119.56(15) |
| C2 | C1 | C8 | 121.36(15) |  | N4 | C22 | C27 | 122.07(14) |
| C6 | C1 | C8 | 120.33(15) |  | C27 | C22 | C23 | 118.36(15) |
| C3 | C2 | C1 | 120.77(17) |  | C24 | C23 | C22 | 120.70(16) |
| C4 | C3 | C2 | 119.98(18) |  | C23 | C24 | C25 | 120.85(15) |
| C5 | C4 | C3 | 120.00(17) |  | C26 | C25 | C24 | 119.56(15) |
| C4 | C5 | C6 | 120.29(18) |  | C25 | C26 | C27 | 121.03(16) |
| C5 | C6 | C1 | 120.67(18) |  | C26 | C27 | C22 | 119.49(14) |
| N1 | C7 | C8 | 109.43(15) |  | C28 | C27 | C22 | 117.74(14) |
| C7 | C8 | C1 | 125.07(16) |  | C28 | C27 | C26 | 122.77(15) |
| C7 | C8 | C9 | 106.20(14) |  | C29 | C28 | C27 | 120.19(15) |
| C9 | C8 | C1 | 128.71(15) |  | C28 | C29 | C30 | 118.47(14) |
| C8 | C9 | C10 | 132.63(14) |  | C28 | C29 | C34 | 125.27(15) |
| C14 | C9 | C8 | 107.36(14) |  | C30 | C29 | C34 | 116.26(14) |
| C14 | C9 | C10 | 119.76(15) |  | N4 | C30 | C29 | 122.58(14) |
| C9 | C10 | C11 | 110.17(14) |  | N4 | C30 | C31 | 118.99(14) |
| C12 | C11 | C10 | 114.07(14) |  | C29 | C30 | C31 | 118.24(14) |
| N2 | C12 | C11 | 118.04(14) |  | C30 | C31 | C32 | 115.45(14) |
| N2 | C12 | C13 | 122.54(15) |  | C33 | C32 | C31 | 110.73(14) |
| C13 | C12 | C11 | 119.26(14) |  | C34 | C33 | C32 | 119.39(14) |
| C12 | C13 | C14 | 115.95(14) |  | C34 | C33 | C36 | 106.93(15) |
| C15 | C13 | C12 | 118.29(15) |  | C36 | C33 | C32 | 133.66(15) |
| C15 | C13 | C14 | 125.75(15) |  | N3 | C34 | C29 | 125.37(14) |
| N1 | C14 | C9 | 108.41(14) |  | N3 | C34 | C33 | 108.99(14) |
| N1 | C14 | C13 | 127.09(14) |  | C33 | C34 | C29 | 125.55(15) |
| C9 | C14 | C13 | 124.48(15) |  | N3 | C35 | C36 | 109.54(15) |
| C13 | C15 | C16 | 119.96(16) |  | C33 | C36 | C37 | 129.72(16) |
| C15 | C16 | C17 | 118.08(15) |  | C35 | C36 | C33 | 106.05(14) |
| C15 | C16 | C21 | 123.18(16) |  | C35 | C36 | C37 | 124.20(16) |
| C21 | C16 | C17 | 118.74(16) |  | C38 | C37 | C36 | 120.03(17) |
| N2 | C17 | C16 | 121.43(15) |  | C42 | C37 | C36 | 122.04(17) |
| N2 | C17 | C18 | 119.05(16) |  | C42 | C37 | C38 | 117.89(17) |
| C18 | C17 | C16 | 119.49(15) |  | C39 | C38 | C37 | 121.0(2) |
| C19 | C18 | C17 | 120.28(17) |  | C40 | C39 | C38 | 120.3(2) |
| C18 | C19 | C20 | 120.47(17) |  | C39 | C40 | C41 | 119.7(2) |
| C21 | C20 | C19 | 120.44(17) |  | C40 | C41 | C42 | 120.6(2) |
| C20 | C21 | C16 | 120.56(17) |  | C37 | C42 | C41 | 120.5(2) |

| **Table 6 Torsion Angles for Npy.** | | | | | | | | | | |
| --- | --- | --- | --- | --- | --- | --- | --- | --- | --- | --- |
| **A** | **B** | **C** | **D** | **Angle/˚** |  | **A** | **B** | **C** | **D** | **Angle/˚** |
| N1 | C7 | C8 | C1 | -178.78(15) |  | N3 | C35 | C36 | C33 | 0.87(19) |
| N1 | C7 | C8 | C9 | -0.54(19) |  | N3 | C35 | C36 | C37 | -177.37(15) |
| N2 | C12 | C13 | C14 | -176.33(15) |  | N4 | C22 | C23 | C24 | -178.66(15) |
| N2 | C12 | C13 | C15 | 4.5(2) |  | N4 | C22 | C27 | C26 | 178.59(15) |
| N2 | C17 | C18 | C19 | 177.08(17) |  | N4 | C22 | C27 | C28 | -1.0(2) |
| C1 | C2 | C3 | C4 | -0.8(3) |  | N4 | C30 | C31 | C32 | -149.84(16) |
| C1 | C8 | C9 | C10 | -7.8(3) |  | C22 | N4 | C30 | C29 | 2.1(2) |
| C1 | C8 | C9 | C14 | 178.12(15) |  | C22 | N4 | C30 | C31 | -172.90(15) |
| C2 | C1 | C6 | C5 | -0.3(3) |  | C22 | C23 | C24 | C25 | -0.7(3) |
| C2 | C1 | C8 | C7 | 134.43(19) |  | C22 | C27 | C28 | C29 | 2.1(2) |
| C2 | C1 | C8 | C9 | -43.4(3) |  | C23 | C22 | C27 | C26 | -0.9(2) |
| C2 | C3 | C4 | C5 | 0.2(3) |  | C23 | C22 | C27 | C28 | 179.49(15) |
| C3 | C4 | C5 | C6 | 0.4(3) |  | C23 | C24 | C25 | C26 | 0.5(3) |
| C4 | C5 | C6 | C1 | -0.3(3) |  | C24 | C25 | C26 | C27 | -0.6(3) |
| C6 | C1 | C2 | C3 | 0.9(3) |  | C25 | C26 | C27 | C22 | 0.8(2) |
| C6 | C1 | C8 | C7 | -43.5(2) |  | C25 | C26 | C27 | C28 | -179.62(16) |
| C6 | C1 | C8 | C9 | 138.69(18) |  | C26 | C27 | C28 | C29 | -177.46(15) |
| C7 | N1 | C14 | C9 | -0.92(18) |  | C27 | C22 | C23 | C24 | 0.9(2) |
| C7 | N1 | C14 | C13 | -179.58(15) |  | C27 | C28 | C29 | C30 | -1.2(2) |
| C7 | C8 | C9 | C10 | 174.01(17) |  | C27 | C28 | C29 | C34 | 179.05(15) |
| C7 | C8 | C9 | C14 | -0.03(18) |  | C28 | C29 | C30 | N4 | -1.0(2) |
| C8 | C1 | C2 | C3 | -177.02(16) |  | C28 | C29 | C30 | C31 | 174.05(15) |
| C8 | C1 | C6 | C5 | 177.62(17) |  | C28 | C29 | C34 | N3 | -10.1(3) |
| C8 | C9 | C10 | C11 | 154.81(17) |  | C28 | C29 | C34 | C33 | 166.24(16) |
| C8 | C9 | C14 | N1 | 0.58(18) |  | C29 | C30 | C31 | C32 | 34.9(2) |
| C8 | C9 | C14 | C13 | 179.28(14) |  | C30 | N4 | C22 | C23 | 178.40(15) |
| C9 | C10 | C11 | C12 | 46.5(2) |  | C30 | N4 | C22 | C27 | -1.1(2) |
| C10 | C9 | C14 | N1 | -174.37(14) |  | C30 | C29 | C34 | N3 | 170.12(15) |
| C10 | C9 | C14 | C13 | 4.3(2) |  | C30 | C29 | C34 | C33 | -13.5(2) |
| C10 | C11 | C12 | N2 | 147.68(16) |  | C30 | C31 | C32 | C33 | -43.4(2) |
| C10 | C11 | C12 | C13 | -36.8(2) |  | C31 | C32 | C33 | C34 | 25.8(2) |
| C11 | C12 | C13 | C14 | 8.4(2) |  | C31 | C32 | C33 | C36 | -155.70(18) |
| C11 | C12 | C13 | C15 | -170.74(15) |  | C32 | C33 | C34 | N3 | 179.28(15) |
| C12 | N2 | C17 | C16 | -1.2(2) |  | C32 | C33 | C34 | C29 | 2.4(3) |
| C12 | N2 | C17 | C18 | -179.47(16) |  | C32 | C33 | C36 | C35 | -179.42(18) |
| C12 | C13 | C14 | N1 | -172.86(15) |  | C32 | C33 | C36 | C37 | -1.3(3) |
| C12 | C13 | C14 | C9 | 8.7(2) |  | C33 | C36 | C37 | C38 | 159.31(17) |
| C12 | C13 | C15 | C16 | -2.2(2) |  | C33 | C36 | C37 | C42 | -23.0(3) |
| C13 | C15 | C16 | C17 | -1.5(2) |  | C34 | N3 | C35 | C36 | -0.64(19) |
| C13 | C15 | C16 | C21 | 178.92(16) |  | C34 | C29 | C30 | N4 | 178.78(15) |
| C14 | N1 | C7 | C8 | 0.91(19) |  | C34 | C29 | C30 | C31 | -6.2(2) |
| C14 | C9 | C10 | C11 | -31.7(2) |  | C34 | C33 | C36 | C35 | -0.78(19) |
| C14 | C13 | C15 | C16 | 178.74(15) |  | C34 | C33 | C36 | C37 | 177.34(17) |
| C15 | C13 | C14 | N1 | 6.2(3) |  | C35 | N3 | C34 | C29 | 177.00(15) |
| C15 | C13 | C14 | C9 | -172.24(16) |  | C35 | N3 | C34 | C33 | 0.13(19) |
| C15 | C16 | C17 | N2 | 3.3(2) |  | C35 | C36 | C37 | C38 | -22.9(3) |
| C15 | C16 | C17 | C18 | -178.41(16) |  | C35 | C36 | C37 | C42 | 154.81(18) |
| C15 | C16 | C21 | C20 | 179.34(17) |  | C36 | C33 | C34 | N3 | 0.41(18) |
| C16 | C17 | C18 | C19 | -1.3(3) |  | C36 | C33 | C34 | C29 | -176.45(15) |
| C17 | N2 | C12 | C11 | 172.53(15) |  | C36 | C37 | C38 | C39 | 177.64(17) |
| C17 | N2 | C12 | C13 | -2.8(2) |  | C36 | C37 | C42 | C41 | -178.03(19) |
| C17 | C16 | C21 | C20 | -0.3(3) |  | C37 | C38 | C39 | C40 | 0.9(3) |
| C17 | C18 | C19 | C20 | 0.3(3) |  | C38 | C37 | C42 | C41 | -0.3(3) |
| C18 | C19 | C20 | C21 | 0.7(3) |  | C38 | C39 | C40 | C41 | -1.1(4) |
| C19 | C20 | C21 | C16 | -0.7(3) |  | C39 | C40 | C41 | C42 | 0.7(4) |
| C21 | C16 | C17 | N2 | -177.06(16) |  | C40 | C41 | C42 | C37 | 0.0(4) |
| C21 | C16 | C17 | C18 | 1.2(3) |  | C42 | C37 | C38 | C39 | -0.1(3) |

| **Table 7 Hydrogen Atom Coordinates (Å×10^4^) and Isotropic Displacement Parameters (Å^2^×10^3^) for Npy.** | | | | |
| --- | --- | --- | --- | --- |
| **Atom** | ***x*** | ***y*** | ***z*** | **U(eq)** |
| H1 | 6278.87 | 1609.95 | 5922.17 | 40 |
| H2 | 3348.63 | 3986.82 | 5374.56 | 44 |
| H3A | 2156.76 | 3954.32 | 5670.46 | 52 |
| H4 | 1791.58 | 1969.25 | 6194.47 | 56 |
| H5 | 2618.95 | 23.38 | 6430 | 55 |
| H6 | 3819.79 | 55.32 | 6155 | 47 |
| H7 | 5234.37 | 1229.56 | 6481.27 | 42 |
| H10A | 3726.64 | 3572.4 | 4368.75 | 43 |
| H10B | 4025.21 | 2272.96 | 3996.53 | 43 |
| H11A | 4385.7 | 4251.47 | 3498.3 | 41 |
| H11B | 4714.19 | 5005.76 | 4228.34 | 41 |
| H15 | 7002.99 | 2052.58 | 5065.46 | 39 |
| H18 | 6560.84 | 4451.07 | 2606.7 | 49 |
| H19 | 7872.68 | 4047.04 | 2685.4 | 55 |
| H20 | 8681.51 | 2967.78 | 3670.35 | 52 |
| H21 | 8172.87 | 2259.89 | 4565.71 | 47 |
| H3 | 5225.65 | 5179.13 | 2729.89 | 39 |
| H23 | 1715.12 | 1022.1 | 1327.75 | 42 |
| H24 | 2107.57 | -1093.55 | 1871.47 | 42 |
| H25 | 3391.67 | -1385.15 | 2580.15 | 40 |
| H26 | 4262.26 | 479.91 | 2752.2 | 40 |
| H28 | 4565.2 | 2976.03 | 2492.51 | 38 |
| H31A | 2473.73 | 6337.82 | 1576.35 | 44 |
| H31B | 2236.37 | 5714.53 | 811.57 | 44 |
| H32A | 3359.41 | 6528.77 | 577.9 | 43 |
| H32B | 3080.95 | 7803.64 | 978.95 | 43 |
| H35 | 5735.43 | 7472.51 | 2545.32 | 41 |
| H38 | 5623.74 | 9837.16 | 2350.1 | 51 |
| H39 | 5859.17 | 12001.84 | 1902.63 | 68 |
| H40 | 5180.56 | 12643.15 | 790.69 | 86 |
| H41 | 4295.56 | 11082.73 | 107.49 | 78 |
| H42 | 4060.45 | 8889.03 | 540.03 | 57 |

| **Table 8 Solvent masks information for Npy.** | | | | | | |
| --- | --- | --- | --- | --- | --- | --- |
| **Number** | **X** | **Y** | **Z** | **Volume** | **Electron count** | **Content** |
| 1 | 0.000 | 0.000 | 0.500 | 118.7 | 38.7 | ? |
| 2 | 0.500 | 0.500 | 0.000 | 118.7 | 38.7 | ? |

**7. References**

1. Frank Nesse, The ORCA program system, *WIREs Computational Molecular Science*, **2012**, *2*, 73–78. DOI: 10.1002/wcms.81.

2. Frank Nesse, The SHARK integral generation and digestion system, *Journal of Computational Chemistry*, **2023**, *44*, 381–396. DOI: 10.1002/jcc.26942.

3. Frank Nesse, Gottfried Olbrich, Efficient use of the resolution of the identity approximation in time-dependent density functional calculations with hybrid density functionals, *Chemical Physics Letters*, **2002**, *362*, 170–178. DOI: 10.1016/S0009-2614(02)01053-9.

4. Stefan Grimme, Jens Antony, Stephan Ehrlich, Helge Krieg, A consistent and accurate ab initio parametrization of density functional dispersion correction (DFT-D) for the 94 elements H-Pu, *The Journal of Chemical Physics*, **2010**, *132*, 154104. DOI: 10.1063/1.3382344.

5. So Hirata, Martin Head-Gordon, Time-dependent density functional theory within the Tamm–Dancoff approximation, *Chemical Physics Letters*, **1999**, *314*, 291–299. DOI: 10.1016/S0009-2614(99)01149-5.

6. Tian Lu, A comprehensive electron wavefunction analysis toolbox for chemists, Multiwfn, *The Journal of Chemical Physics*, **2024**, *161*, 082503. DOI: 10.1063/5.0216272.
